# Crystal structure and Hirshfeld surface analysis of 5,5-di­fluoro-10-[5-(tri­methyl­sil­yl)furan-2-yl]-5*H*-4λ^4^,5λ^4^-di­pyrrolo­[1,2-*c*:2′,1′-*f*][1,3,2]di­aza­borinine

**DOI:** 10.1107/S2056989025004888

**Published:** 2025-06-06

**Authors:** Dmitriy M. Shchevnikov, Alexandra G. Kutasevich, Victor N. Khrustalev, Narmina A. Guliyeva, Khudayar I. Hasanov, Mehmet Akkurt, Gizachew Mulugeta Manahelohe

**Affiliations:** a RUDN University, 6 Miklukho-Maklaya St., Moscow 117198, Russian Federation; b Zelinsky Institute of Organic Chemistry of RAS, 4, 7 Leninsky Prospect, 119991 Moscow, Russian Federation; c Department of Chemical Engineering, Baku Engineering University, Hasan Aliyev, str. 120, Baku, Absheron AZ0101, Azerbaijan; d Azerbaijan Medical University, Scientific Research Centre (SRC), A. Kasumzade St. 14. AZ 1022, Baku, Azerbaijan; e Department of Physics, Faculty of Sciences, Erciyes University, 38039 Kayseri, Türkiye; f Department of Chemistry, University of Gondar, PO Box 196, Gondar, Ethiopia; Katholieke Universiteit Leuven, Belgium

**Keywords:** crystal structure, C—H⋯π inter­actions, π–π inter­actions, Hirshfeld surface analysis

## Abstract

In the title compound, C_16_H_17_BF_2_N_2_OSi, the mol­ecular conformation is consolidated by an intra­molecular C—H⋯O hydrogen bond, forming an *S*(6) motif. In the crystal, pairs of mol­ecules are connected by C—H⋯π and π–π inter­actions [centroid-to-centroid distance = 3.6155 (8) Å] between the furan rings. These dimers are linked by π–π inter­actions [centroid-to-centroid distance = 3.4041 (9) Å] between similar five-membered rings of the twelve-membered ring system, forming ribbons along the *a*-axis direction. As a result, the van der Waals inter­actions between the ribbons provide crystal cohesion. Hirshfeld surface analysis indicates that H⋯H (48.6%), F⋯H/H⋯F (19.8%) and C⋯H/H⋯C (19.0%) inter­actions make the most significant contributions to the crystal packing.

## Abstract 2

In the title compound, C_16_H_17_BF_2_N_2_OSi, the mol­ecular conformation is stabilized by an intra­molecular C—H⋯O hydrogen bond. In the crystal, mol­ecules are connected by C—H⋯π and π–π inter­actions, forming ribbons along the *a*-axis direction.

## 1. Chemical context

**Figure 1:**
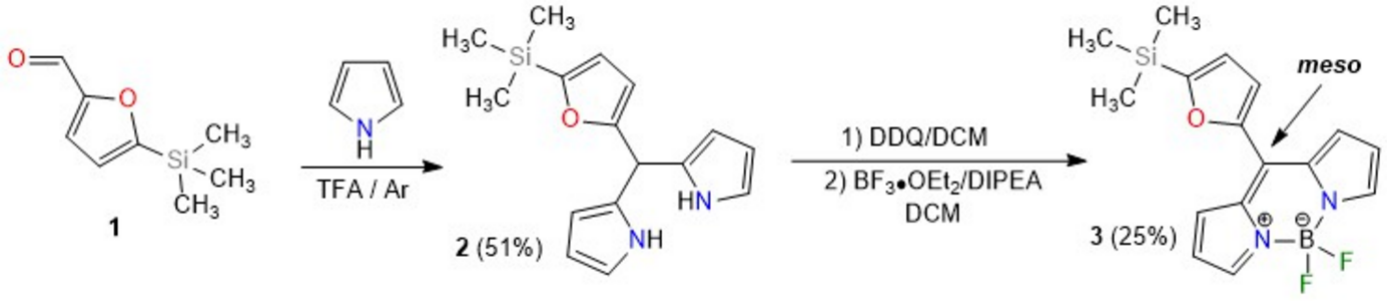
Synthesis of 5,5-di­fluoro-10-[5-(tri­methyl­sil­yl)furan-2-yl]-5*H*-4λ^4^,5λ^4^-di­pyrrolo­[1,2-*c*:2′,1′-*f*][1,3,2]di­aza­borinine.

4,4-Di­fluoro-4-bora-3a,4a-di­aza-*s*-indacene (BODIPY) complexes are strongly UV-absorbing small mol­ecules with high quantum yields. Since their discovery in 1968 by Treibs and Kreuzer (Treibs & Kreuzer, 1968 ▸), BODIPYs have been established in several research areas. They are relatively insensitive to the polarity and pH of their environment and are reasonably stable to physiological conditions. They are acknowledged as valuable fluorescent tags with applications in bioimaging and have been investigated as part of photodynamic therapy. They have been used as efficient photosensitizers, laser dyes, fluorescent switches, photocatalysts, labeling reagents, photocages, and chemosensors (Loudet & Burgess, 2007 ▸; Ulrich *et al.*, 2008 ▸; Turksoy *et al.*, 2019 ▸; Boens *et al.*, 2019 ▸; Poddar & Misra, 2020 ▸; Agazzi *et al.*, 2019 ▸; Velásquez *et al.*, 2019 ▸). The electronic properties of BODIPY can be changed by replacing the six-membered *meso*-aryl substituent (the *meso*-position is marked on Fig. 1 ▸) with five-membered aromatic heterocycles such as pyrrole, thio­phene, furan, and seleno­phene. Indeed these heterocycle rings are small and may align with the plane of the BODIPY moiety and be involved in its delocalization, leading to further modification of the electronic properties of BODIPY. It has previously been shown that replacing the six-membered aryl group with five-membered heterocycles significantly alters the electronic properties, which are reflected in their structure, and the spectroscopic and electrochemical properties compared to *meso*-aryl BODIPY (Kim *et al.*, 2010 ▸; Sharma *et al.*, 2016 ▸). Moreover, decoration of BODIPY with non-covalent bond donor or acceptor sites can be used as a synthetic strategy in catalysis (Gurbanov *et al.*, 2022 ▸; Kopylovich *et al.*, 2012 ▸; Mahmudov & Pombeiro, 2023 ▸), crystal engineering (Askerov *et al.*, 2020 ▸; Pronina *et al.*, 2024 ▸) and material chemistry (Khalilov, 2021 ▸; Polyanskii *et al.*, 2019 ▸). Continuing our research on the chemistry of five-membered heterocycle-substituted dipyrrolmethanes and their complexes (BODIPY; Sadikhova *et al.*, 2024 ▸), we used 5-(tri­methyl­sil­yl)furan-5-carbaldehyde (Zubkov *et al.*, 2016 ▸), which, when reacted with pyrrole, gave the target dipyrrolmethane **2** in 51% yield. In the next step, *meso*-tri­methyl­silylfuryl dipyrrolmethane **2** was oxidized with DDQ (2,3-di­chloro-5,6-di­cyano­benzo­quinone) in CH_2_Cl_2_ for 30 min, the resulting dipyrrolmethene was neutralized with DIPEA (diiso­propyl­ethyl­amine), and the BF_2_ complexation was carried out by the addition of BF_3_·(OEt)_2_. Column chromatographic purification on silica afforded the *meso*-furyl BODIPY **3** in 25% yield (Fig. 1 ▸).
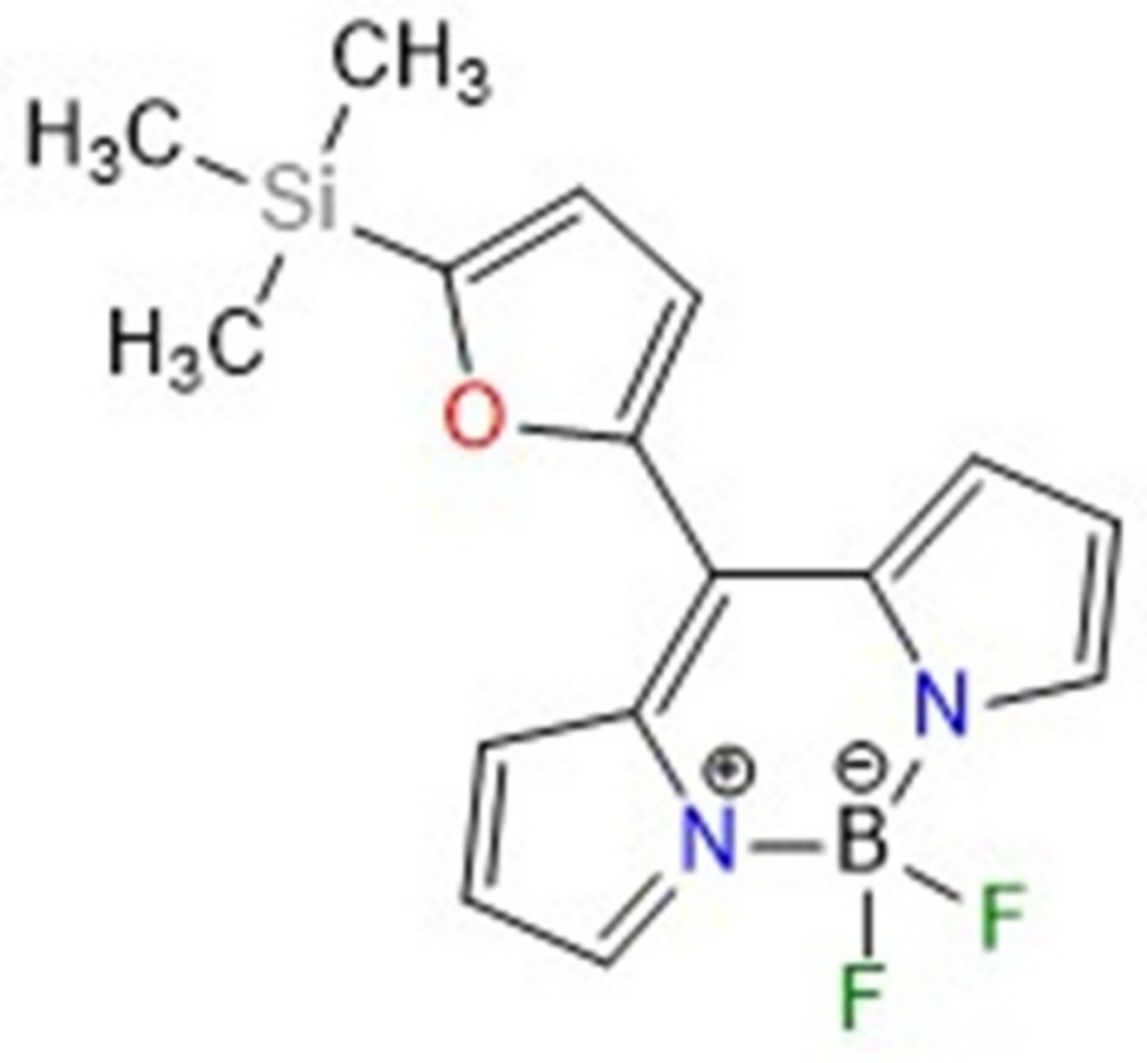


## 2. Structural commentary

**Figure 2:**
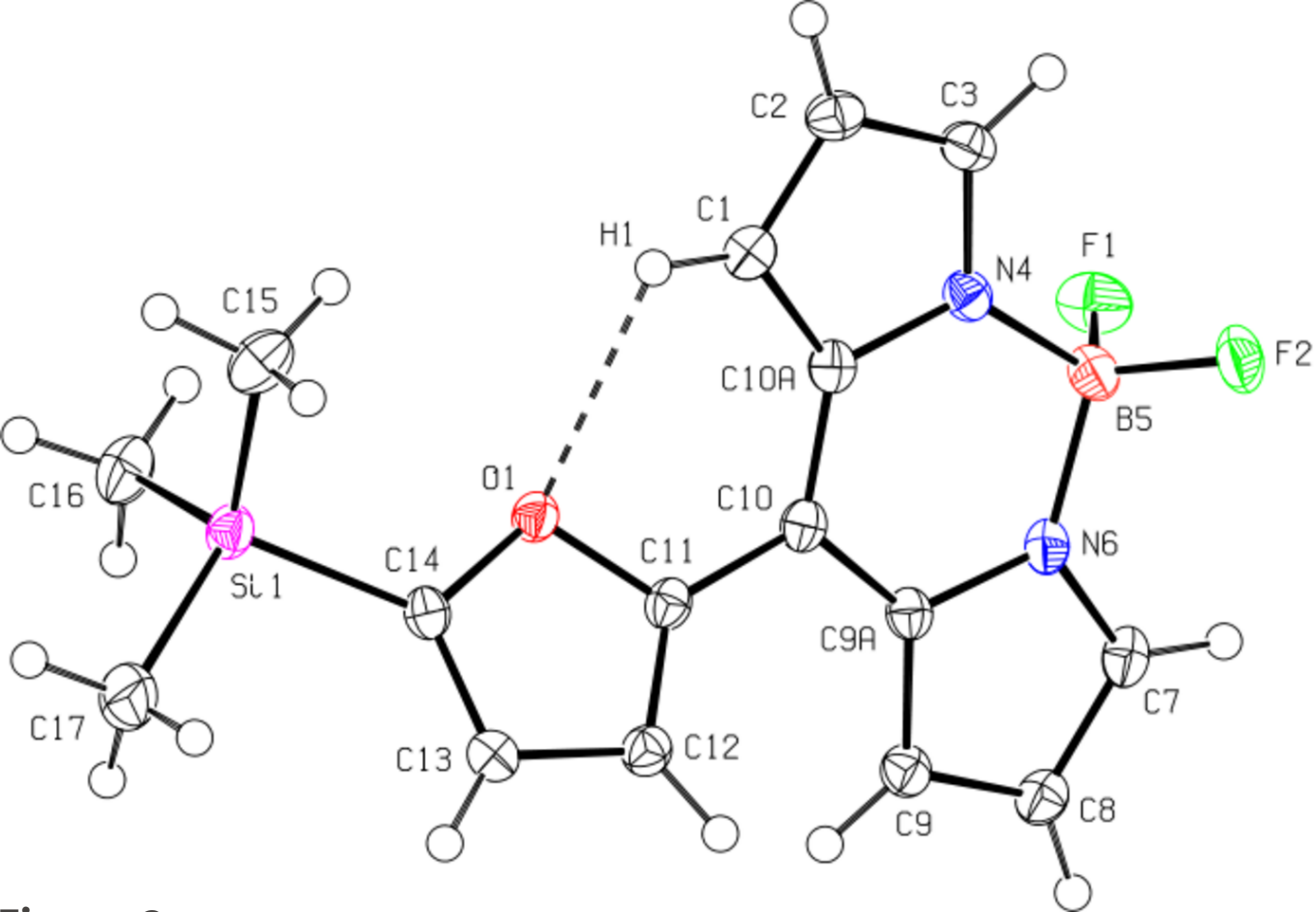
Mol­ecular structure of the title compound showing the atomic labelling. Displacement ellipsoids are drawn at the 50% probability level.

The mol­ecular conformation of the title compound is consolidated by an intra­molecular C1—H1⋯O1 hydrogen bond, forming an *S*(6) motif (Fig. 2 ▸, Table 1 ▸; Bernstein *et al.*, 1995 ▸). The mean plane of the twelve-membered ring system (C1–C3/N4/B5/N6/C7–C9/C9*A*/C10/C10*A*; r.m.s. deviation of fitted atoms = 0.0267 Å) makes a dihedral angle of 33.34 (6)° with the furan ring (O1/C11–C14). The torsion angles F1—B5—N4—C3 and F2—B5—N6—C7 are −64.52 (19) and −59.55 (19)°, respectively. The distances of atoms F1 and F2 to the mean plane of the twelve-membered ring system are −1.253 (1) and 1.004 (1) Å, respectively. In other words, F1 and F2 are on the opposite side of the ring system. All geometric parameters are normal and consistent with those of related compounds listed in the section *Database survey*.

## 3. Supra­molecular features and Hirshfeld surface analysis

**Figure 3:**
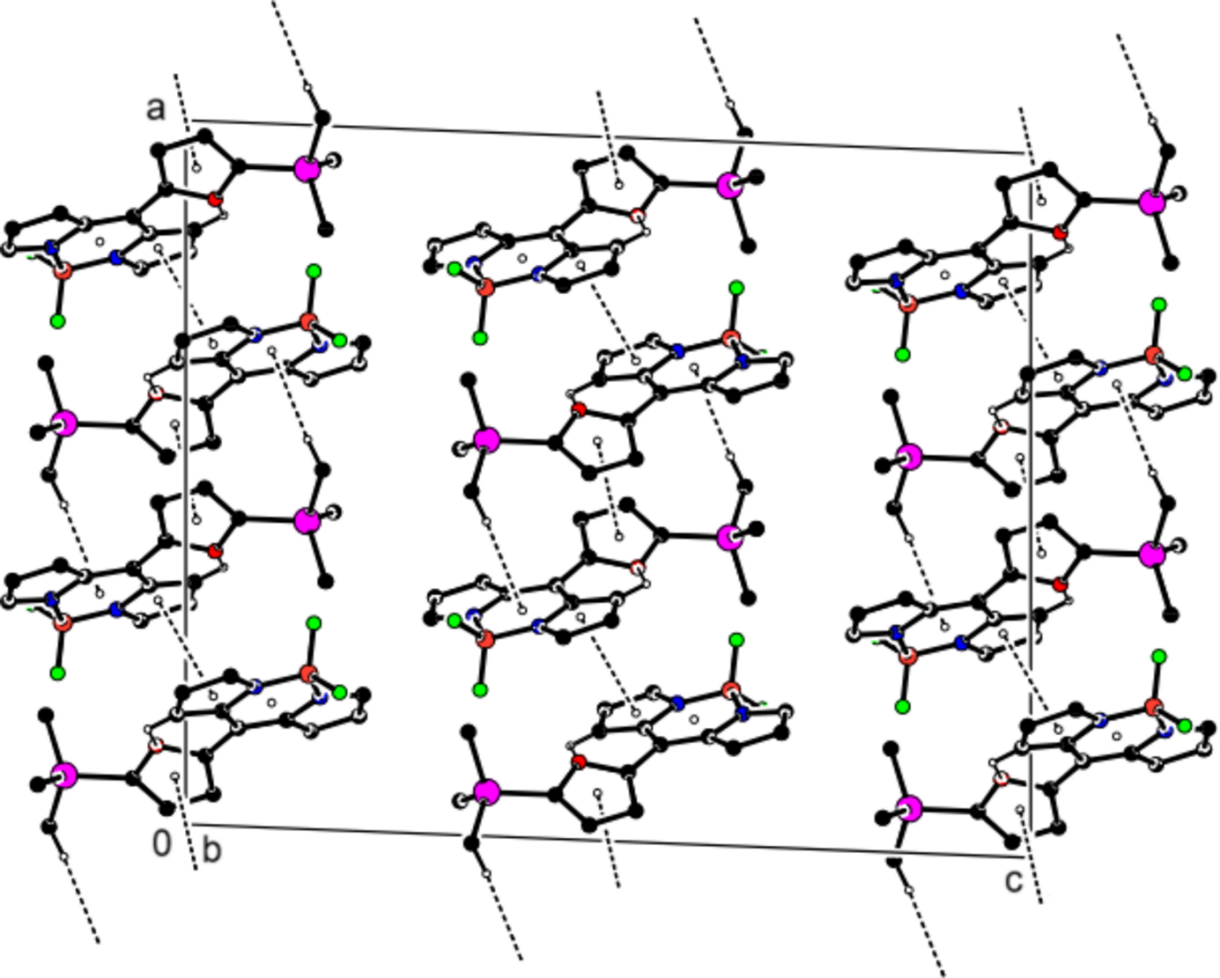
Crystal packing of the title compound viewed along the *b* axis showing the C—H⋯O, C—H⋯π and π–π inter­actions (dashed lines). H atoms not involved in hydrogen bonding have been omitted.

In the crystal, pairs of mol­ecules are connected by C—H⋯π inter­actions [C17—H17*A*⋯*Cg*4^i^; C17⋯*Cg*4^*i*^ = 3.906 (2) Å, H17*A*⋯*Cg*4^i^ = 2.93 Å, C17—H17*A*⋯*Cg*4^i^ = 172°; symmetry code: (i) *x* − 1, *y* − 1, *z* − 1; *Cg*4 is the centroid of the six-membered central ring B5/N4/N6/C9*A*/C10/C10*A* of the large ring system] and π–π inter­actions between the furan rings (O1/C11–C14) [*Cg*1⋯*Cg*1^i^ = 3.6155 (8) Å, slippage = 1.063 Å; symmetry code: (i) 1 − *x*, 1 − *y*, 1 − *z*; *Cg*1 is the centroid of the furan ring]. These dimers are linked by π–π inter­actions [*Cg2*⋯*Cg*2^ii^ = 3.4041 (9) Å, slippage = 0.696 Å; symmetry code: (ii) 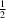 − *x*, 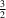 − *y*, 1 − *z*; *Cg*2 is the centroid of the five-membered ring N4/C1–C3/C10*A*] between the five-membered rings (N4/C1–C3/C10*A*) of the twelve-membered ring system, forming ribbons along the *a*-axis direction (Table 1 ▸; Fig. 3 ▸). van der Waals inter­actions between the ribbons provide further crystal cohesion.

**Figure 4:**
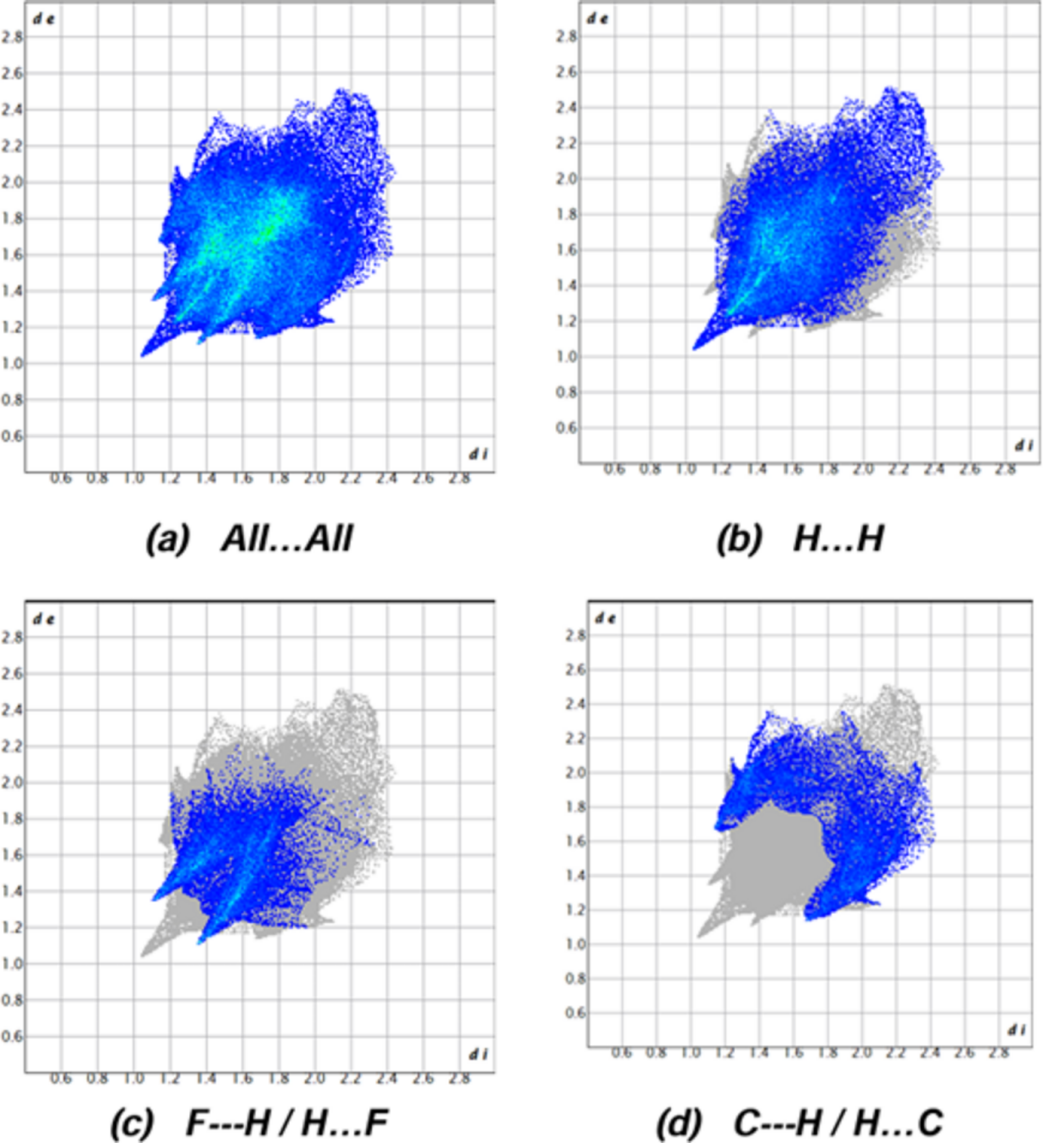
The two-dimensional fingerprint plots for the title compound showing (*a*) all inter­actions, and those delineated into (*b*) H⋯H, (*c*) F⋯H/H⋯F and (*d*) C⋯H/H⋯C inter­actions. The *d*_i_ and *d*_e_ values are the closest inter­nal and external distances (in Å) from given points on the Hirshfeld surface.

Hirshfeld surfaces were generated for the mol­ecule of the title compound using *Crystal Explorer 17.5* (Spackman *et al.*, 2021 ▸). Fingerprint plots (Fig. 4 ▸) reveal that while H⋯H inter­actions (48.6%) make the largest contributions to the surface contacts (Tables 1 ▸ and 2 ▸), F⋯H/H⋯F (19.8%) and C⋯H/H⋯C (19.0%) inter­actions are also important. Other, less notable inter­actions are C⋯C (4.8%), O⋯H/H⋯O (3.4%), N⋯H/H⋯N (3.2%), N⋯C/C⋯N (0.6%), O⋯C/C⋯O (0.4%) and F⋯C/C⋯F (0.3%).

## 4. Database survey

A search in the Cambridge Structural Database (CSD, version 6.00, update April 2025; Groom *et al.*, 2016 ▸) for *5,5-di­fluoro-10-(furan-2-yl)-5H-4l4,5l4-di­pyrrolo­[1,2-c:2′,1′-f][1,3,2] di­aza­borinine* (twelve-membered ring moiety with a furan substituent) gives twelve hits, *viz.* GATDIQ (Khan & Ravikanth, 2012 ▸), GATDOW (Khan & Ravikanth, 2012 ▸), KETDAQ (Jun *et al.*, 2012*a* ▸), NARSAC (Khan *et al.*, 2012 ▸), NARSEG (Khan *et al.*, 2012 ▸), ROZGEU (Zhao *et al.*, 2015 ▸), ROZHAR (Zhao *et al.*, 2015 ▸), ROZHEV (Zhao *et al.*, 2015 ▸), UKANUQ (Kim *et al.*, 2010 ▸), UKANUQ01 (Khan *et al.*, 2012 ▸), ULAQOP (Sharma *et al.*, 2016 ▸) and XELDAV (Jun *et al.*, 2012*b* ▸).

GATDIQ, GATDOW, ROZGEU and ROZHAR crystallize in the triclinic space group *P*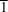. KETDAQ and ULAQOP crystallize in the ortho­rhom­bic space groups *Pbca* and *Pna*2_1_, respectively. NARSAC, ROZHEV, UKANUQ and UKANUQ01 crystallize in the monoclinic space group *P*2_1_/*c*, while NARSEG and XELDAV crystallize in the monoclinic space groups *P*2_1_/*n* and *C*2/*c*, respectively.

The dihedral angle between the two ring systems (furan substituent and twelve-membered ring moiety) varies between 25.93 (10) and 88.13 (14)°, and is influenced by the substitution pattern and mol­ecular environment. In GATDIG, with two independent mol­ecules in the asymmetric unit, the dihedral angles are 33.31 (10) and 33.85 (9)°, in GATDOW 44.4 (5)°, in KETDAQ 88.13 (14)°, in ROZHAR 84.82 (8)°, in ROZHEV 78.02 (9)°, in ULAQOP 31.24 (16)°, in XELDAV 75.60 (13)°, and in NARSAC, with two independent mol­ecules in the asymmetric unit, 29.4 (2) and 32.2 (2)°, respectively. In NARSEG, the furan ring is disordered over two positions, the dihedral angles are 83.0 (3) and 36.9 (2)°, respectively. In UKANUQ, with two independent mol­ecules in the asymmetric unit, the dihedral angles are 26.59 (16) and 26.92 (17)°, with similar values for UKANUQ01 [26.65 (10) and 25.93 (10)°].

In KETDAQ, ROZGEU and ROZHEV, C—H⋯F intra­molecular inter­actions are observed, while in ROZHAR there are C—H⋯S and C—H⋯F inter­actions. In the remaining compounds, there are intra­molecular C—H⋯O hydrogen bonds involving the O atom of the furan ring. Additionally, in NARSEG and ULAQOP, besides C—H⋯O, there are also C—H⋯F inter­actions, and in XELDAV, intra­molecular C—H⋯S inter­actions are present as well.

## 5. Synthesis and crystallization

5-(Tri­methyl­sil­yl)furan-2-carbaldehyde **1** (100 mg, 0.6 mmol) was dissolved in excess of pyrrole (1 mL, 15 mmol) at room temperature under argon. Tri­fluoro­acetic acid (TFA, 4.6 µl, 0.06 mmol) was added dropwise, and the reaction was stirred for 1 h (TLC control). Then Et_3_N (50 µL) was added to pH ∼7. The reaction mixture was poured into water (30 mL) and extracted with ethyl acetate (3 × 10 mL). The target product was purified by column chromatography (eluent: ethyl acetate/hexane 1:10), yield 51%, 86.9 mg (0.306 mmol), dark-brown oil. IR (KBr), ν (cm^−1^): 3398 (NH). ^1^H NMR (700.2 MHz, CDCl_3_) (*J*, Hz): δ 8.10 (*br.s*, 2H, NH), 6.72–6.71 (*m*, 2H, H Pyr), 6.57 (*d*, *J* = 3.1, 1H, H Fur), 6.17–6.16 (*m*, 2H, H Pyr), 6.09 (*d*, *J* = 3.1, 1H, H Fur), 6.00–5.99 (*m*, 2H, H Pyr), 5.55 (*s*, 1H, CH), 0.27 (*s*, 9H, CH_3_). ^13^C{^1^H} NMR (176.1 MHz, CDCl_3_): δ 159.9, 158.4, 130.3 (2C), 120.4, 117.4 (2C), 108.2 (2C), 107.1, 106.7 (2C), 37.9, −1.57 (3C). MS (ESI) *m*/*z*: [*M* + H]^+^ 285.

Dipyrrolmethane **2** (80 mg, 0.3 mmol) was dissolved in dry DCM (5 ml), after 2,3-di­chloro-5,6-di­cyano­benzo­quinone (DDQ, 192 mg, 0.6 mmol) was added; the reaction mixture was stirred for 30 min (TLC control), poured into water (30 mL) and extracted with DCM (3 × 10 mL). The organic layer was dried with anhydrous Na_2_SO_4_, concentrated *in vacuo* and the residue was dissolved in dry DCM (5 ml) without further purification. Boron trifluoride etherate (700 µl, 6 mmol) and an equal volume of diiso­propyl­ethyl­amine (DIPEA, 700 µl, 4 mmol) were added. The solution was stirred under room temperature for 1 h (TLC control) and then poured into water (30 mL), extracted with DCM (3 × 10 mL) and washed with saturated Na_2_CO_3_ (3 × 10 mL). The organic layer was dried with anhydrous Na_2_SO_4_, the target product **3** was purified by column chromatography (eluent: ethyl acetate/hexane 1:10); red crystals, yield 25%, 24.8 mg (0.075 mmol), m.p. 393–395 K. Single crystals of the title compound were grown from a mixture of ethyl acetate/hexane. IR (KBr), ν (cm^−1^): 1566, 1539, 1412, 1386, 1119, 1081, 841. ^1^H NMR (700.2 MHz, CDCl_3_) (*J*, Hz): δ 7.89 (*br.s*, 2H, H Pyr), 7.47 (*br.d*, *J* = 4.3, 2H, H Pyr), 7.21 (*d*, *J* = 3.3, 1H, H Fur), 6.89 (*d*, *J* = 3.3, 1H, H Fur), 6.00-5.99 (*br.dd*, *J* = 4.3, 1.2, 2H, H Pyr), 0.40 (*s*, 9H, CH_3_). ^13^C{^1^H} NMR (176.1 MHz, CDCl_3_): δ 168.5, 152.4, 142.7 (2C), 132.2, 130.4 (2C), 122.5 (2C), 121.0 (2C), 118.1 (2C), −1.81 (3C). ^19^F{^1^H} NMR (658.8 MHz, CDCl_3_): −145.8 (*q*, *J* = 28.6). MS (ESI) *m*/*z*: [*M*]^+^ 330.

## 6. Refinement

Crystal data, data collection and structure refinement details are summarized in Table 3 ▸. All C-bound H atoms were positioned geometrically (C—H = 0.95 and 0.98 Å) and included as riding contributions with isotropic displacement parameters fixed at 1.2*U*_eq_(C) (1.5 for methyl groups).

## Supplementary Material

Crystal structure: contains datablock(s) I. DOI: 10.1107/S2056989025004888/vm2314sup1.cif

Structure factors: contains datablock(s) I. DOI: 10.1107/S2056989025004888/vm2314Isup2.hkl

CCDC reference: 2455268

Additional supporting information:  crystallographic information; 3D view; checkCIF report

## Figures and Tables

**Table 1:** Hydrogen-bond geometry (Å, °) *Cg*4 is the centroid of the B5/N4/N6/C9*A*/C10/C10*A* ring.

*D*—H⋯*A*	*D*—H	H⋯*A*	*D*⋯*A*	*D*—H⋯*A*
C1—H1⋯O1	0.95	2.46	2.9210 (18)	110
C17—H17*A*⋯*Cg*4^i^	0.98	2.93	3.906 (2)	172

Symmetry code: (i) 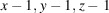.

**Table 2:** Summary of short inter­atomic contacts (Å) in the title compound

Contact	Distance	Symmetry operation
F2⋯H7	2.56	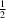 − *x*, 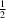 + *y*, 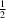 − *z*
H15*A*⋯F1	2.55	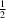 − *x*, 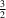 − *y*, 1 − *z*
H16*B*⋯F1	2.65	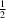 − *x*, 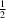 − *y*, 1 − *z*
F2⋯H8	2.70	*x*, 1 + *y*, *z*
F2⋯H16*A*	2.83	*x*, 1 − *y*, − 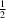 + *z*
H13⋯C1	2.91	1 − *x*, 1 − *y*, 1 − *z*
H13⋯H13	2.55	1 − *x*, −*y*, 1 − *z*
H17*C*⋯H17*C*	2.27	1 − *x*, *y*, 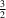 − *z*

**Table 3:** Experimental details

Crystal data	
Chemical formula	C_16_H_17_BF_2_N_2_OSi
*M*_r_	330.21
Crystal system, space group	Monoclinic, *C*2/*c*
Temperature (K)	100
*a*, *b*, *c* (Å)	19.2621 (2), 7.20455 (11), 23.1309 (3)
β (°)	92.2617 (12)
*V* (Å^3^)	3207.48 (7)
*Z*	8
Radiation type	Cu *K*α
μ (mm^−1^)	1.52
Crystal size (mm)	0.30 × 0.15 × 0.03

Data collection	
Diffractometer	Rigaku XtaLAB Synergy-S, HyPix-6000HE area-detector
Absorption correction	Multi-scan (*CrysAlis PRO*; Rigaku OD, 2021 ▸)
*T*_min_, *T*_max_	0.704, 0.955
No. of measured, independent and observed [*I* > 2σ(*I*)] reflections	21486, 3453, 3119
*R*_int_	0.047
(sin θ/λ)_max_ (Å^−1^)	0.639

Refinement	
*R*[*F*^2^ > 2σ(*F*^2^)], *wR*(*F*^2^), *S*	0.039, 0.102, 1.06
No. of reflections	3453
No. of parameters	211
H-atom treatment	H-atom parameters constrained
Δρ_max_, Δρ_min_ (e Å^−3^)	0.41, −0.28

Computer programs: *CrysAlis PRO* (Rigaku OD, 2021 ▸), *SHELXT2016/6* (Sheldrick, 2015*a* ▸), *SHELXL2016/6* (Sheldrick, 2015*b* ▸, *ORTEP-3 for Windows* (Farrugia, 2012 ▸) and *PLATON* (Spek, 2020 ▸).

## supplementary crystallographic information

### 5,5-Difluoro-10-[5-(trimethylsilyl)furan-2-yl]-5H-4λ4,5λ4-dipyrrolo[1,2-c:2',1'-f][1,3,2]diazaborinine Crystal data

**Table:**

C_16_H_17_BF_2_N_2_OSi	*F*(000) = 1376
*M**_r_* = 330.21	*D*_x_ = 1.368 Mg m^−^^3^
Monoclinic, *C*2/*c*	Cu *K*α radiation, λ = 1.54184 Å
*a* = 19.2621 (2) Å	Cell parameters from 10600 reflections
*b* = 7.20455 (11) Å	θ = 3.8–78.8°
*c* = 23.1309 (3) Å	µ = 1.52 mm^−^^1^
β = 92.2617 (12)°	*T* = 100 K
*V* = 3207.48 (7) Å^3^	Plate, red
*Z* = 8	0.30 × 0.15 × 0.03 mm

### 5,5-Difluoro-10-[5-(trimethylsilyl)furan-2-yl]-5H-4λ4,5λ4-dipyrrolo[1,2-c:2',1'-f][1,3,2]diazaborinine Data collection

**Table:**

Rigaku XtaLAB Synergy-S, HyPix-6000HE area-detector diffractometer	3119 reflections with *I* > 2σ(*I*)
Radiation source: micro-focus sealed X-ray tube	*R*_int_ = 0.047
φ and ω scans	θ_max_ = 80.1°, θ_min_ = 3.8°
Absorption correction: multi-scan (CrysAlisPro; Rigaku OD, 2021)	*h* = −23→24
*T*_min_ = 0.704, *T*_max_ = 0.955	*k* = −6→8
21486 measured reflections	*l* = −29→29
3453 independent reflections	

### 5,5-Difluoro-10-[5-(trimethylsilyl)furan-2-yl]-5H-4λ4,5λ4-dipyrrolo[1,2-c:2',1'-f][1,3,2]diazaborinine Refinement

**Table:**

Refinement on *F*^2^	Primary atom site location: difference Fourier map
Least-squares matrix: full	Secondary atom site location: difference Fourier map
*R*[*F*^2^ > 2σ(*F*^2^)] = 0.039	Hydrogen site location: inferred from neighbouring sites
*wR*(*F*^2^) = 0.102	H-atom parameters constrained
*S* = 1.06	*w* = 1/[σ^2^(*F*_o_^2^) + (0.0491*P*)^2^ + 3.5578*P*] where *P* = (*F*_o_^2^ + 2*F*_c_^2^)/3
3453 reflections	(Δ/σ)_max_ = 0.001
211 parameters	Δρ_max_ = 0.41 e Å^−^^3^
0 restraints	Δρ_min_ = −0.28 e Å^−^^3^

### 5,5-Difluoro-10-[5-(trimethylsilyl)furan-2-yl]-5H-4λ4,5λ4-dipyrrolo[1,2-c:2',1'-f][1,3,2]diazaborinine Special details

**Table:**

Experimental. CrysAlisPro 1.171.43.143a (Rigaku OD, 2021). Empirical absorption correction using spherical harmonics, implemented in SCALE3 ABSPACK scaling algorithm.
Geometry. All esds (except the esd in the dihedral angle between two l.s. planes) are estimated using the full covariance matrix. The cell esds are taken into account individually in the estimation of esds in distances, angles and torsion angles; correlations between esds in cell parameters are only used when they are defined by crystal symmetry. An approximate (isotropic) treatment of cell esds is used for estimating esds involving l.s. planes.

### 5,5-Difluoro-10-[5-(trimethylsilyl)furan-2-yl]-5H-4λ4,5λ4-dipyrrolo[1,2-c:2',1'-f][1,3,2]diazaborinine Fractional atomic coordinates and isotropic or equivalent isotropic displacement parameters (Å^2^)

**Table:**

	*x*	*y*	*z*	*U*_iso_*/*U*_eq_	
Si1	0.43746 (2)	0.28241 (6)	0.64368 (2)	0.02098 (13)	
F1	0.20739 (5)	0.77309 (15)	0.34832 (4)	0.0325 (2)	
F2	0.30413 (6)	0.91603 (13)	0.31804 (4)	0.0316 (2)	
O1	0.38976 (5)	0.42058 (15)	0.53488 (4)	0.0188 (2)	
C1	0.34470 (7)	0.8007 (2)	0.51052 (6)	0.0204 (3)	
H1	0.367195	0.754235	0.544828	0.025*	
C2	0.31332 (8)	0.9727 (2)	0.50384 (7)	0.0221 (3)	
H2	0.310273	1.066288	0.532573	0.027*	
C3	0.28701 (8)	0.9823 (2)	0.44676 (7)	0.0215 (3)	
H3	0.262664	1.085502	0.430357	0.026*	
N4	0.30104 (6)	0.82473 (18)	0.41836 (5)	0.0188 (3)	
B5	0.27939 (9)	0.7816 (2)	0.35470 (7)	0.0220 (3)	
N6	0.31188 (6)	0.59102 (18)	0.34116 (5)	0.0187 (3)	
C7	0.30625 (8)	0.5016 (2)	0.29015 (6)	0.0215 (3)	
H7	0.285003	0.551110	0.255735	0.026*	
C8	0.33624 (8)	0.3250 (2)	0.29513 (6)	0.0217 (3)	
H8	0.339222	0.235224	0.265252	0.026*	
C9	0.36070 (8)	0.3063 (2)	0.35182 (6)	0.0203 (3)	
H9	0.383070	0.200181	0.368307	0.024*	
C9A	0.34630 (7)	0.4743 (2)	0.38085 (6)	0.0180 (3)	
C10	0.36032 (7)	0.5326 (2)	0.43820 (6)	0.0178 (3)	
C10A	0.33712 (7)	0.7069 (2)	0.45689 (6)	0.0181 (3)	
C11	0.40153 (7)	0.4126 (2)	0.47664 (6)	0.0181 (3)	
C12	0.45553 (7)	0.2958 (2)	0.46751 (6)	0.0198 (3)	
H12	0.474219	0.265782	0.431281	0.024*	
C13	0.47852 (7)	0.2275 (2)	0.52241 (6)	0.0196 (3)	
H13	0.515816	0.143437	0.529818	0.023*	
C14	0.43752 (7)	0.3042 (2)	0.56265 (6)	0.0181 (3)	
C15	0.45176 (9)	0.5176 (3)	0.67517 (7)	0.0297 (4)	
H15A	0.416726	0.603296	0.658774	0.044*	
H15B	0.498208	0.561639	0.666085	0.044*	
H15C	0.447867	0.511655	0.717248	0.044*	
C16	0.35303 (9)	0.1850 (3)	0.66563 (7)	0.0304 (4)	
H16A	0.354477	0.165501	0.707587	0.046*	
H16B	0.344600	0.066182	0.645926	0.046*	
H16C	0.315546	0.271840	0.654924	0.046*	
C17	0.51091 (10)	0.1209 (3)	0.66193 (8)	0.0396 (5)	
H17A	0.553132	0.165821	0.644222	0.059*	
H17B	0.499544	−0.003364	0.647107	0.059*	
H17C	0.518595	0.115323	0.704025	0.059*	

### 5,5-Difluoro-10-[5-(trimethylsilyl)furan-2-yl]-5H-4λ4,5λ4-dipyrrolo[1,2-c:2',1'-f][1,3,2]diazaborinine Atomic displacement parameters (Å^2^)

**Table:**

	*U*^11^	*U*^22^	*U*^33^	*U*^12^	*U*^13^	*U*^23^
Si1	0.0198 (2)	0.0267 (2)	0.0164 (2)	0.00247 (16)	0.00028 (14)	0.00248 (15)
F1	0.0254 (5)	0.0357 (6)	0.0355 (5)	0.0059 (4)	−0.0103 (4)	−0.0078 (4)
F2	0.0547 (6)	0.0184 (5)	0.0213 (4)	−0.0038 (4)	−0.0022 (4)	0.0043 (4)
O1	0.0192 (5)	0.0213 (5)	0.0158 (5)	0.0014 (4)	0.0004 (4)	0.0009 (4)
C1	0.0191 (7)	0.0228 (8)	0.0194 (7)	−0.0020 (5)	0.0007 (5)	−0.0011 (6)
C2	0.0208 (7)	0.0208 (8)	0.0248 (7)	−0.0017 (6)	0.0028 (5)	−0.0044 (6)
C3	0.0211 (7)	0.0181 (7)	0.0255 (7)	0.0009 (6)	0.0024 (5)	0.0000 (6)
N4	0.0187 (6)	0.0173 (6)	0.0206 (6)	−0.0011 (5)	0.0008 (4)	0.0015 (5)
B5	0.0253 (8)	0.0186 (8)	0.0216 (8)	−0.0002 (6)	−0.0034 (6)	0.0012 (6)
N6	0.0217 (6)	0.0185 (6)	0.0159 (5)	−0.0038 (5)	−0.0006 (4)	0.0020 (5)
C7	0.0242 (7)	0.0231 (8)	0.0169 (6)	−0.0059 (6)	−0.0009 (5)	0.0017 (6)
C8	0.0246 (7)	0.0210 (8)	0.0194 (7)	−0.0051 (6)	0.0019 (5)	−0.0024 (6)
C9	0.0221 (7)	0.0182 (7)	0.0207 (7)	−0.0024 (5)	0.0011 (5)	0.0001 (6)
C9A	0.0184 (6)	0.0171 (7)	0.0184 (7)	−0.0031 (5)	0.0002 (5)	0.0015 (5)
C10	0.0161 (6)	0.0192 (7)	0.0183 (6)	−0.0039 (5)	0.0021 (5)	0.0017 (5)
C10A	0.0165 (6)	0.0194 (7)	0.0184 (6)	−0.0028 (5)	0.0001 (5)	0.0014 (5)
C11	0.0195 (6)	0.0190 (7)	0.0159 (6)	−0.0032 (5)	0.0007 (5)	−0.0003 (5)
C12	0.0202 (7)	0.0198 (7)	0.0195 (7)	−0.0015 (5)	0.0024 (5)	−0.0002 (5)
C13	0.0184 (6)	0.0184 (7)	0.0217 (7)	−0.0008 (5)	−0.0008 (5)	0.0001 (6)
C14	0.0173 (6)	0.0186 (7)	0.0182 (6)	−0.0016 (5)	−0.0014 (5)	0.0022 (5)
C15	0.0274 (8)	0.0377 (10)	0.0239 (8)	−0.0045 (7)	0.0016 (6)	−0.0065 (7)
C16	0.0317 (8)	0.0351 (10)	0.0249 (8)	−0.0066 (7)	0.0059 (6)	0.0014 (7)
C17	0.0413 (10)	0.0517 (12)	0.0257 (8)	0.0206 (9)	0.0009 (7)	0.0094 (8)

### 5,5-Difluoro-10-[5-(trimethylsilyl)furan-2-yl]-5H-4λ4,5λ4-dipyrrolo[1,2-c:2',1'-f][1,3,2]diazaborinine Geometric parameters (Å, º)

**Table:**

Si1—C16	1.8602 (17)	C8—C9	1.382 (2)
Si1—C15	1.8604 (19)	C8—H8	0.9500
Si1—C17	1.8675 (18)	C9—C9A	1.417 (2)
Si1—C14	1.8809 (15)	C9—H9	0.9500
F1—B5	1.390 (2)	C9A—C10	1.407 (2)
F2—B5	1.384 (2)	C10—C10A	1.406 (2)
O1—C11	1.3760 (17)	C10—C11	1.453 (2)
O1—C14	1.3841 (17)	C11—C12	1.361 (2)
C1—C2	1.385 (2)	C12—C13	1.416 (2)
C1—C10A	1.415 (2)	C12—H12	0.9500
C1—H1	0.9500	C13—C14	1.361 (2)
C2—C3	1.397 (2)	C13—H13	0.9500
C2—H2	0.9500	C15—H15A	0.9800
C3—N4	1.345 (2)	C15—H15B	0.9800
C3—H3	0.9500	C15—H15C	0.9800
N4—C10A	1.3961 (19)	C16—H16A	0.9800
N4—B5	1.546 (2)	C16—H16B	0.9800
B5—N6	1.546 (2)	C16—H16C	0.9800
N6—C7	1.3450 (19)	C17—H17A	0.9800
N6—C9A	1.3932 (19)	C17—H17B	0.9800
C7—C8	1.400 (2)	C17—H17C	0.9800
C7—H7	0.9500		
			
C16—Si1—C15	110.75 (8)	N6—C9A—C9	107.48 (12)
C16—Si1—C17	111.45 (9)	C10—C9A—C9	131.89 (14)
C15—Si1—C17	112.33 (9)	C10A—C10—C9A	120.31 (13)
C16—Si1—C14	109.78 (7)	C10A—C10—C11	121.03 (13)
C15—Si1—C14	107.96 (7)	C9A—C10—C11	118.62 (13)
C17—Si1—C14	104.30 (7)	N4—C10A—C10	120.19 (13)
C11—O1—C14	107.27 (11)	N4—C10A—C1	107.49 (13)
C2—C1—C10A	107.47 (13)	C10—C10A—C1	132.29 (14)
C2—C1—H1	126.3	C12—C11—O1	109.51 (12)
C10A—C1—H1	126.3	C12—C11—C10	132.48 (13)
C1—C2—C3	106.87 (14)	O1—C11—C10	117.84 (12)
C1—C2—H2	126.6	C11—C12—C13	106.83 (13)
C3—C2—H2	126.6	C11—C12—H12	126.6
N4—C3—C2	110.34 (14)	C13—C12—H12	126.6
N4—C3—H3	124.8	C14—C13—C12	107.62 (13)
C2—C3—H3	124.8	C14—C13—H13	126.2
C3—N4—C10A	107.83 (12)	C12—C13—H13	126.2
C3—N4—B5	125.69 (13)	C13—C14—O1	108.76 (12)
C10A—N4—B5	126.47 (13)	C13—C14—Si1	132.11 (11)
F2—B5—F1	109.38 (13)	O1—C14—Si1	119.11 (10)
F2—B5—N6	110.22 (13)	Si1—C15—H15A	109.5
F1—B5—N6	110.46 (13)	Si1—C15—H15B	109.5
F2—B5—N4	110.87 (13)	H15A—C15—H15B	109.5
F1—B5—N4	109.90 (13)	Si1—C15—H15C	109.5
N6—B5—N4	105.98 (12)	H15A—C15—H15C	109.5
C7—N6—C9A	107.99 (13)	H15B—C15—H15C	109.5
C7—N6—B5	125.70 (13)	Si1—C16—H16A	109.5
C9A—N6—B5	126.05 (12)	Si1—C16—H16B	109.5
N6—C7—C8	110.13 (13)	H16A—C16—H16B	109.5
N6—C7—H7	124.9	Si1—C16—H16C	109.5
C8—C7—H7	124.9	H16A—C16—H16C	109.5
C9—C8—C7	106.90 (13)	H16B—C16—H16C	109.5
C9—C8—H8	126.5	Si1—C17—H17A	109.5
C7—C8—H8	126.5	Si1—C17—H17B	109.5
C8—C9—C9A	107.48 (14)	H17A—C17—H17B	109.5
C8—C9—H9	126.3	Si1—C17—H17C	109.5
C9A—C9—H9	126.3	H17A—C17—H17C	109.5
N6—C9A—C10	120.63 (13)	H17B—C17—H17C	109.5
			
C10A—C1—C2—C3	0.08 (16)	C3—N4—C10A—C10	−178.23 (13)
C1—C2—C3—N4	−0.19 (17)	B5—N4—C10A—C10	3.0 (2)
C2—C3—N4—C10A	0.21 (17)	C3—N4—C10A—C1	−0.15 (16)
C2—C3—N4—B5	179.00 (13)	B5—N4—C10A—C1	−178.93 (13)
C3—N4—B5—F2	56.52 (19)	C9A—C10—C10A—N4	−1.1 (2)
C10A—N4—B5—F2	−124.92 (15)	C11—C10—C10A—N4	176.29 (12)
C3—N4—B5—F1	−64.52 (19)	C9A—C10—C10A—C1	−178.63 (15)
C10A—N4—B5—F1	114.04 (16)	C11—C10—C10A—C1	−1.2 (2)
C3—N4—B5—N6	176.11 (13)	C2—C1—C10A—N4	0.04 (16)
C10A—N4—B5—N6	−5.32 (19)	C2—C1—C10A—C10	177.79 (15)
F2—B5—N6—C7	−59.55 (19)	C14—O1—C11—C12	−0.46 (16)
F1—B5—N6—C7	61.44 (19)	C14—O1—C11—C10	−176.34 (12)
N4—B5—N6—C7	−179.57 (13)	C10A—C10—C11—C12	−143.13 (16)
F2—B5—N6—C9A	126.96 (14)	C9A—C10—C11—C12	34.3 (2)
F1—B5—N6—C9A	−112.05 (15)	C10A—C10—C11—O1	31.60 (19)
N4—B5—N6—C9A	6.94 (19)	C9A—C10—C11—O1	−150.95 (13)
C9A—N6—C7—C8	0.33 (16)	O1—C11—C12—C13	0.06 (17)
B5—N6—C7—C8	−174.14 (13)	C10—C11—C12—C13	175.12 (15)
N6—C7—C8—C9	0.42 (17)	C11—C12—C13—C14	0.38 (17)
C7—C8—C9—C9A	−0.98 (16)	C12—C13—C14—O1	−0.67 (17)
C7—N6—C9A—C10	179.29 (13)	C12—C13—C14—Si1	−178.96 (12)
B5—N6—C9A—C10	−6.3 (2)	C11—O1—C14—C13	0.70 (15)
C7—N6—C9A—C9	−0.93 (15)	C11—O1—C14—Si1	179.25 (10)
B5—N6—C9A—C9	173.51 (13)	C16—Si1—C14—C13	−119.49 (15)
C8—C9—C9A—N6	1.19 (16)	C15—Si1—C14—C13	119.69 (15)
C8—C9—C9A—C10	−179.06 (15)	C17—Si1—C14—C13	0.03 (18)
N6—C9A—C10—C10A	2.7 (2)	C16—Si1—C14—O1	62.36 (13)
C9—C9A—C10—C10A	−177.04 (14)	C15—Si1—C14—O1	−58.47 (13)
N6—C9A—C10—C11	−174.78 (12)	C17—Si1—C14—O1	−178.13 (12)
C9—C9A—C10—C11	5.5 (2)		

### 5,5-Difluoro-10-[5-(trimethylsilyl)furan-2-yl]-5H-4λ4,5λ4-dipyrrolo[1,2-c:2',1'-f][1,3,2]diazaborinine Hydrogen-bond geometry (Å, º)

*Cg*4 is the centroid of the B5/N4/N6/C9*A*/C10/C10*A* ring.

**Table:**

*D*—H···*A*	*D*—H	H···*A*	*D*···*A*	*D*—H···*A*
C1—H1···O1	0.95	2.46	2.9210 (18)	110
C17—H17*A*···*Cg*4^i^	0.98	2.93	3.906 (2)	172

Symmetry code: (i) −*x*+1, −*y*+1, −*z*+1.
